# Effects of Transition Element Additions on the Interfacial Interaction and Electronic Structure of Al(111)/6H-SiC(0001) Interface: A First-Principles Study

**DOI:** 10.3390/ma14030630

**Published:** 2021-01-29

**Authors:** Changqing Wang, Weiguang Chen, Jingpei Xie

**Affiliations:** 1 Collaborative Innovation Center of Nonferrous Metals of Henan Province, College of Materials Science and Engineering, Henan University of Science and Technology, Luoyang 471023, China; cqw@lit.edu.cn; 2 Department of Mathematics and Physics, Luoyang Institute of Science and Technology, Luoyang 471023, China; 3 School of Physics and Electronic Engineering, Zhengzhou Normal University, Zhengzhou 450044, China; 15937915186@163.com

**Keywords:** first-principles, metal/ceramic interfaces, Al matrix composite, adhesion energy, transition element additions

## Abstract

In this work, the effects of 20 transition element additions on the interfacial adhesion energy and electronic structure of Al(111)/6H-SiC(0001) interfaces have been studied by the first-principles method. For pristine Al(111)/6H-SiC(0001) interfaces, both Si-terminated and C-terminated interfaces have covalent bond characteristics. The C-terminated interface has higher binding energy, which is mainly due to the stronger covalent bond formed by the larger charge transfer between C and Al. The results show that the introduction of many transition elements, such as 3d transitional group Mn, Fe, Co, Ni, Cu, Zn and 4d transitional group Tc, Ru, Rh, Pd, Ag, can improve the interfacial adhesion energy of the Si-terminated Al(111)/6H-SiC(0001) interface. However, for the C-terminated Al(111)/6H-SiC(0001) interface, only the addition of Co element can improve the interfacial adhesion energy. Bader charge analysis shows that the increase of interfacial binding energy is mainly attributed to more charge transfer.

## 1. Introduction

SiC particle reinforced aluminum matrix composites have been widely used in aerospace, automobile and other industries owing to their good physical and chemical properties [1,2,3]. Because the load is transferred through the interface, SiC/Al interface plays an important role on the properties of composites [4]. In order to improve the wettability and adhesion of Al/SiC interfaces, adding transition elements into Al matrix has become one of the most common methods to prepare high-performance composites [5].

In experiments, many researchers have studied the wetting and bonding behavior of Al/SiC interfaces [6,7]. By means of the static drop technique in high vacuum, Laurent et al. have researched the wetting kinetics of Al/SiC interfaces [8]. They found that the addition of Sn can improve the wettability of Al/SiC interface, which is due to the decrease of surface tension of Al, while the addition of Cu can reduce the interaction between Al and SiC, thus reducing the wettability. Similar experiments show that the wettability of Al/SiC system can be improved by adding a small amount of Mg into Al matrix [9]. In addition, the experimental results show that Cu, Si and Mg can reduce the amount of Al_4_C_3_ formed on the interface and improve the Al/SiC interface reaction [10,11]. The effect of Si addition on wettability of Al melt may be attributed to the segregation of Si on the interface and the strengthened bond between Si and SiC [11].

In recent decades, first-principles calculations based on density functional theory (DFT) have become one of the most powerful tools for studying metal-ceramic interfaces at atomic and even electronic levels [12,13,14,15]. It can give quantitatively the atomic and electronic structures at the interface and the influence of alloying elements on the stability of the interface [16,17,18]. The results show that strong covalent bonds can be formed at the metal-ceramic interface and the bonding strength of the interface can be improved by adding alloying elements to the metal matrix. SiC is a typical compound with polytypic structure. The number of Si-C stacking layers in a complete repeatable cell determines the type of SiC. According to the classification of SiC structure, H and C denote hexagonal and cubic cell, respectively. The number of stacking layers in the cell is represented by numbers. 4H and 6H indicate that there are four and six Si-C layers in a hexagonal symmetric cell, respectively. 3C represents that there are three Si-C layers in a cubic cell. In earlier years, Al/SiC interfaces have been investigated by quantum chemistry methods [19] and ab-initio calculations [20,21]. Recently, the structural and mechanical properties of the Al(111)/6H-SiC(0001) [22,23,24] and Al (100)/6H–SiC(0001) [25] interfaces have been studied using the first-principles method. All these studies suggest that the strong bonding of SiC/Al interface is attributed to the formation of covalent bonds. Apart from these, effects of alloying element additions on interfacial adhesion properties of Al(111)/4H-SiC(0001) [26] and Al(111)/3C-SiC(111) [27] interfaces have been studied by the first-principles method. However, a systematic theoretical study on effects of transition metal adhesions on the Al(111)/6H-SiC(0001) interfacial properties have been rarely reported.

In this paper, first-principles calculations were performed to investigate effects of twenty transition element additions on the interfacial interaction and electronic structure of Al(111)/6H-SiC(0001) interface. The results show that the interfacial bonding energy of the Si-terminated Al(111)/6H-SiC(0001) interface can be improved by introducing 3d transition group elements, such as Mn, Fe, Co, Ni, Cu, Zn, and 4d transition group elements, such as Tc, Ru, Rh, Pd, Ag. However, for the C-terminal one, only adding the Co element can improve the interfacial bonding energy. Bader charge analysis shows that the interfacial binding energy is closely related to atomic charge transfer. Our calculated results can give a profound understanding of the mechanism of alloying elements that improve the adhesive strength of Al(111)/6H-SiC(0001) interfaces.

## 2. Details of Calculation Methods

**Figure 1:**
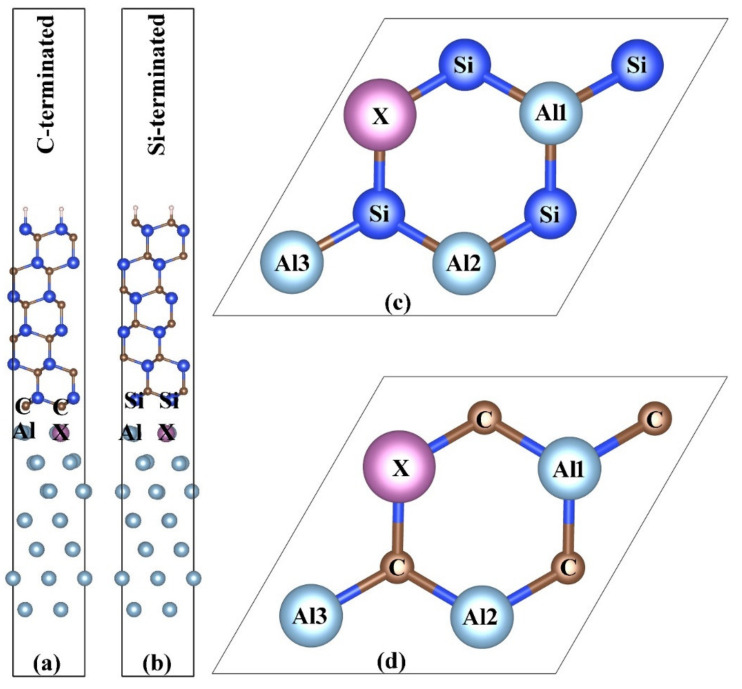
The 2 × 1 × 1 supercell model of Al(111)/6H-SiC(0001) interface. (**a**) Side view of the C-terminated interface; (**b**) side view of the Si-terminated interface; (**c**) top view of the C-terminated interface; (**d**) top view of the Si-terminated interface. Light-blue, brown, blue and violet spheres represent Al, C, Si and impurity atoms, respectively.

In this work, first-principles calculations were carried out by the Vienna ab-initio simulation package (VASP) code [28,29]. Total energy and electronic structure calculations were performed with the projector augmented-wave (PAW) [30,31] method. The generalized gradient approximation (GGA) with the Perdew–Burke–Ernzerhof (PBE) [32] approach was used to describe the exchange correlation functional. The cut-off energy value of wave functions was set to 600 eV. The energy calculations were conducted in the first irreducible Brillouin zone with a Γ-centered 15 × 15 × 1 Monkhorst–Pack (MP) grid [33]. The convergence criteria for electron and ion relaxation have been set as 10^−5^ and 10^−4^ eV, respectively. Meanwhile, for interface calculations, the force tolerance of each atom was set as 10^−2^ eV/Å. According to previous studies [19,20,21,22,23,27], the binding energy of the Al(111)/6H-SiC(0001) is the highest when the C(Si) atom is directly above the Al atom. Therefore, we only studied this configuration here. We tested that the surface energy of Al (111) slab with six Al atomic layers converged to a certain value, and that of SiC (0001) slab with five SiC layers converged to a certain value. By increasing the number of atomic layers of Al and SiC, the interfacial adhesion energy was almost unchanged. In addition, the recent work also presents that the surface energy of the Al (111) slab with seven atomic layers can converge to a certain value [26]. As shown in Figure 1, a 2 × 2 × 1 supercell was used to do all calculations in this research. The supercell consisted of seven Al atomic layers and six SiC atomic layers. A 2 × 2 Al slab along the [–110] and [–101] base vectors matched a 2 × 2 SiC slab. The lattice constants of Al and SiC were 2.859 Å and 3.095 Å, respectively. Thus, the lattice mismatch was about 7.6%. The softer aluminum matrix was stretched along two basis vectors to form a coherent interface with the harder SiC. In order to eliminate the influence between adjacent supercells, a vacuum layer of no less than 20 Å was left in the *z* direction. The whole supercell was relaxed to release the internal stress. One of the interfacial Al atoms was replaced by a transition metal atom X. In this way, the interface doping concentration was 25%, while the bulk doping was only 3.57%.

## 3. Results and Discussion

### 3.1. Pristine Interfaces

**Figure 2:**
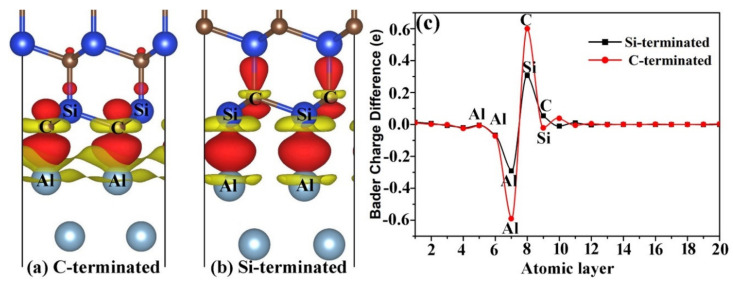
Charge density difference of the Al(111)/6H-SiC(0001) interfaces. Red and yellow denote charge enrichment and deficiency, respectively. The isosurfaces are set to 0.003 e/Å^3^ (**a**) the C-terminated interface; (**b**) the Si-terminated interface; (**c**) Bader charge difference diagram of different atomic layers of the Al(111)/6H-SiC(0001) interfaces. The positive and negative values represent the gain and loss charges, respectively.

The atomic and electronic structures of Al(111)/6H-SiC(0001) interface have been given in detail in our previous studies [22,23]. In order to compare with the results of the following doping interfaces, we further studied the interfacial charge transfer. Figure 2 shows the charge density difference of the Al(111)/6H-SiC(0001) interfaces. The charge density difference is defined as

(1) $$\rho_{diff}=\rho_{Al(111)/SiC(0001)}-\rho_{Al(111)}-\rho_{SiC(0001)}$$

where $\rho_{Al(111)/SiC(0001)}$, $\rho_{Al(111)}$ and $\rho_{SiC(0001)}$ are the charge density of the Al(111)/SiC(0001) interface system, the isolated Al(111) and SiC(0001) slabs, respectively. According to this definition, a positive value represents charge enrichment and a negative value represents charge dissipation. It can be seen from the Figure 2a,b that the atomic charges at the interface were rearranged regardless of Si- or C-terminated interface. Charge transfer occurred between Al matrix and SiC. Some charges from Al matrix and SiC accumulated at the interface. At the C-terminated interface, a covalent bond was formed between C and Al atoms. In the same way, covalent bonds were formed between Si and Al atoms at the Si-terminated interface. Moreover, the length of C–Al bond was about 1.99 Å, which was much smaller than that of Si–Al bond about 2.53 Å. The shorter the bond length is, the greater the binding energy was, and there should be more charge transfer. The adhesion energy of C-terminated interface was about 3.90 J/m^2^, which was indeed larger than that of Si-terminated interface, 2.93 J/m^2^. The interfacial adhesion energy was defined as the energy required to form the interface per unit area. It is expressed by the formula:

(2) $$E_{ad}=\frac{E_{Al(111)}+E_{SiC(0001)}-E_{Al(111)/SiC(0001)}}{A}$$

where $E_{Al(111)}$, $E_{SiC(0001)}$ and $E_{Al(111)/SiC(0001)}$ represent the energy of the Al(111) slab, the SiC(0001) slab and the Al(111)/6H-SiC(0001) interface, respectively. A is the area of the interface.

Detailed analysis of atomic charges may help to understand bonding properties such as bond strength. In this paper, we will focus on the atomic net charge distributions according to Bader analysis [34,35]. The Bader charge difference of each atom in the interface system is defined as

(3) $$Q_{diff}=Q_{Al(111)/SiC(0001)}-Q_{Al(111)}{-Q}_{SiC(0001)}$$

where $Q_{Al(111)/SiC(0001)}$, $Q_{Al(111)}$ and $Q_{SiC(0001)}$ represent the Bader charge of the corresponding atom in the Al(111)/SiC(0001) interface, the Al(111) and the SiC(0001) slab. Thus, $Q_{diff}>0$ indicates that the charge of the atom increases and $Q_{diff}<0$ indicates that the charge of the atom decreases. The Bader charge difference of each atom in each atomic layer is shown in Figure 2c. It can be seen from the figure that only the atomic charge at the interface changed significantly. For the C-terminated Al(111)/SiC(0001) interface, the Al atom lost charge and the C atom gained charge. Similarly, for the Si-terminated interface, the Al atom lost the charge and the Si atom gained the charge. Therefore, it can be seen that when SiC and Al formed the interface, the electrons in Al matrix transferred to SiC. More importantly, there were more transferred charges, about 0.6 e/atom, at the C-terminated Al(111)/SiC(0001) interface than the Si-terminated one, about 0.3 e/atom. It is the reason why the adhesion energy of the C-terminated Al(111)/SiC(0001) interface was larger than that of the Si-terminated one.

### 3.2. Doping Interfaces

**Figure 3:**
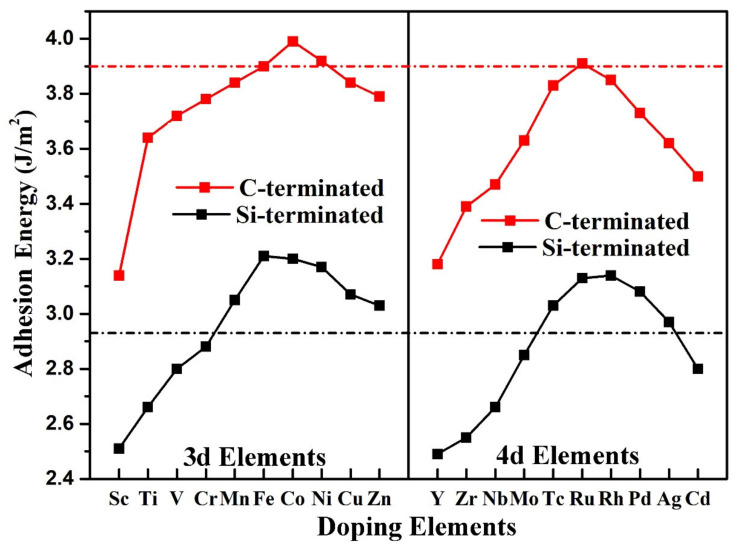
Adhesion energies of the Al-X(111)/SiC(0001) interface with different doping elements. The red and black dashed lines represent adhesion energies of pristine C-terminated and Si-terminated Al(111)/6H-SiC(0001) interfaces, respectively.

The effects of alloying elements on the interfacial interaction were systematically studied by replacing an Al atom with a transition metal atom. For all 3d and 4d transition families, a total of 20 transition metal elements were considered in this work. The adhesion energy is a very important mechanical parameter to describe the interface bonding characteristics. Similar to the pristine interface, it can be defined as

(4) $$E_{ad}=\frac{E_{Al-X(111)}+E_{SiC(0001)}-E_{Al-X(111)/SiC(0001)}}{A}$$

where $E_{Al-X(111)}$, $E_{SiC(0001)}$ and $E_{Al-X(111)/SiC(0001)}$ denote the energy of the Al-X(111) slab, the SiC(0001) slab and the Al-X(111)/6H-SiC(0001) interface, respectively. A is the area of the interface. X stands for a doping element. Adhesion energies of the Al-X(111)/SiC(0001) interface with different doping elements have been shown in Figure 3. For comparison, the adhesion work of pure Al (111)/SiC (0001) interface is also shown as the dashed lines in the figure. The red dashed line represents the adhesion work of the C-terminated Al(111)/SiC(0001) interface, and the black dashed line represents the adhesive work of the Si-terminated Al(111)/SiC(0001) interface. It can be seen from the figure that, similar to the pristine interface, the adhesion work of C-terminated interface was greater than that of Si-terminated interface for the same doping element. For the C-terminated interface, only the Co element doped in the Al matrix could significantly improve the interfacial adhesion energy. It is mainly because the bond strength of C–Co was greater than that of C–Al. However, for the Si-terminated interface, many elements, such as Mn, Fe, Co, Ni, Cu, Zn 3d transition elements and Tc, Ru, Rh, Pd, Ag 4d transition elements, could improve the interfacial adhesion energy. It is mainly due to the greater bonding strength of these elements with Si than that of Al and Si. It can be concluded that the introduction of transition metal elements into Al matrix was mainly to improve the binding energy of the Si-terminated interface. The same conclusion was obtained for Cu doped at the Al(111)/4H-SiC(0001) interface [26] and Mg doped at the Al(111)/3C-SiC(111) interface [27]. That is to say the doping of Cu and Mg into the Al matrix could increase the bonding of the Si-terminated interface, but decrease the binding of the C-terminated interface.

**Figure 4:**
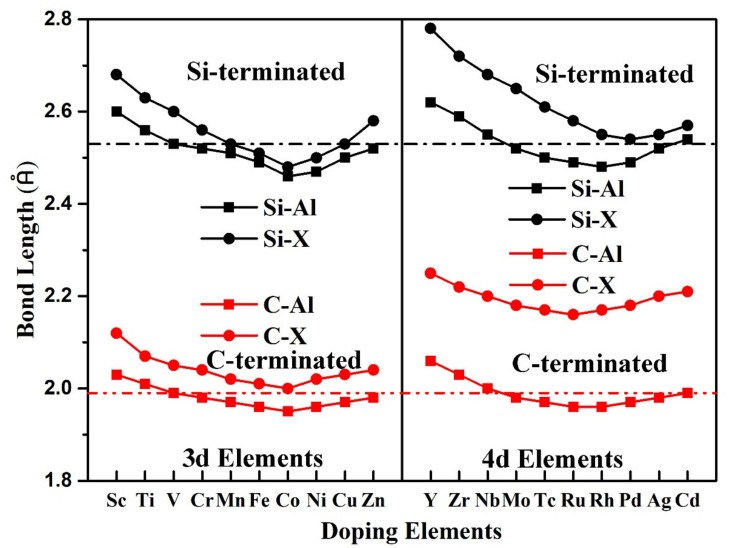
Bond lengths at the Al-X/SiC(0001) interface with different doping elements. The red and black dashed lines represent bond lengths of pristine C-terminated and Si-terminated Al(111)/6H-SiC(0001) interfaces, respectively.

Figure 4 shows bond lengths at the Al-X/SiC(0001) interface with different doping elements. For comparison, the bond lengths of the pristine Al(111)/SiC(0001) interface are also shown as the dashed lines in the figure. The red dashed line represents the C-Al bond length at the C-terminated Al(111)/SiC(0001) interface, and the black dashed line represents the Si-Al bond length at the Si-terminated Al(111)/SiC(0001) interface. The black solid circles and squares represent the bond lengths of Si–X and Si–Al at the Si-terminated Al-X(111)/SiC(0001) interface, respectively. The red solid circles and squares represent the bond lengths of C–X and C–Al at the C-terminated Al–X(111)/SiC(0001) interface, respectively. X stands for a doping element. As can be seen from Figure 4, the bond length at the C-terminated interface, whether C–Al or C–X, was shorter than that at the Si-terminated interface, just like that at the pristine interface. It showed again that the bonding strength of C-terminated interface was higher than that of Si-terminated interface. For all transition elements X, the length of C–X bond was longer than that of C–Al bond at the C-terminated interface. The same was true for the Si-terminated interface. This is mainly because the atomic radius of the transition metal element X is longer than that of Al. Due to the introduction of transition elements, such as Mn, Fe, Co, Ni, Cu, Zn, Tc, Ru, Rh, Pd, Ag, the length of Si–Al bond at the Si-terminated Al-X(111)/6H-SiC(0001) interface was shorter than that of pristine Al (111)/6H-SiC(0001) interface. The shorter the bond length, the stronger the bond. In order to facilitate researchers to access the relevant data, all results of bond lengths and adhesion energies at the Al-X(111)/6H-SiC (0001) interface are summarized in Table 1 and Table 2.

**Figure 5:**
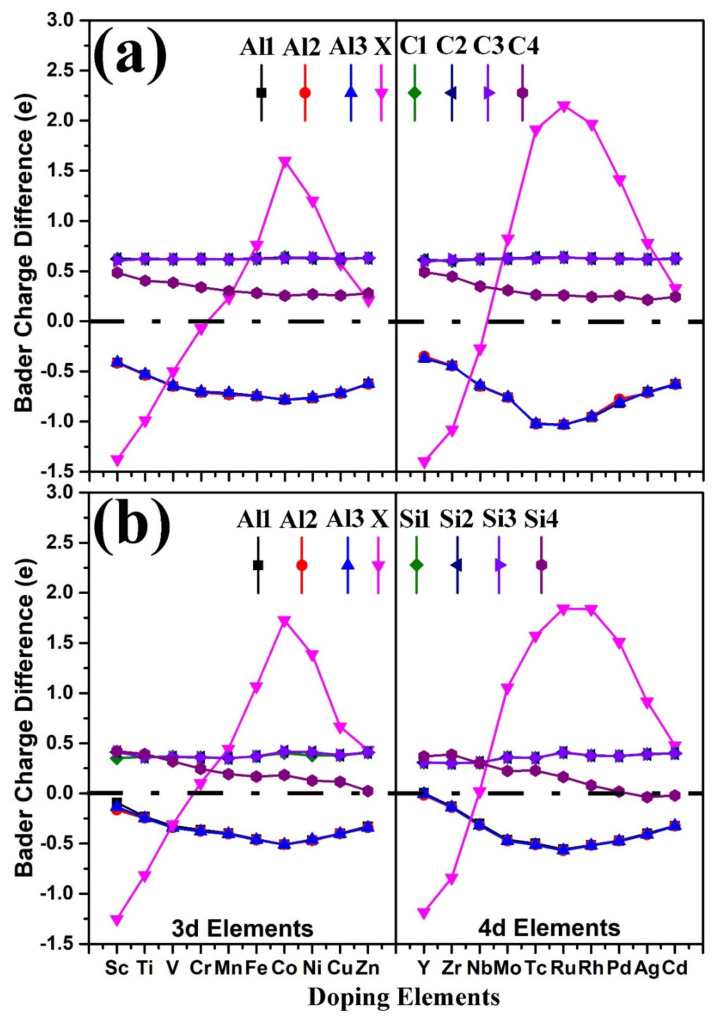
Bader charge difference of each atom at the Al-X(111)/6H-SiC(0001) interface. The positive and negative values represent the gain and loss of charges, respectively. The serial numbers of the eight atoms at the interface are the same as those in Figure 1. (**a**) the C-terminated interface; (**b**) the Si-terminated interface.

The charge of each atom at the interface was calculated by Bader analysis. By analyzing the charge of atom, we can know the transfer of charge. Bader charge difference of each atom at the Al-X(111)/6H-SiC(0001) interface has been shown in Figure 5. The positive and negative values represent the gain and loss of charges, respectively. The serial numbers of the eight atoms at the interface are the same as those in Figure 1. Al1, Al2, Al3 and X denote three Al and doping atoms, respectively. C1, C2, C3, C4 and Si1, Si2, Si3, Si4 represent four C and Si atoms, respectively. As can be seen from the Figure 5, the Bader charge difference of each nonmetal atom (whether C1, C2, C3, C4 atom of the C-terminated interface or Si1, Si2, Si3, Si4 atom of the Si-terminated interface) at the interface was positive. The Bader charge difference of each Al atom at the interface was negative. These results showed that nonmetal atoms gained charges and Al atoms lost charges. Consequently, covalent bonds were formed between metal and nonmetal atoms at the interface. Whether 3d or 4d transition elements were introduced at the Al-X(111)/6H-SiC(0001) interface, as shown in Figure 5, the Bader charge difference of C1, C2, C3 carbon atoms had no obvious change. Only the Bader charge difference of the C4 atom, which was above the doping atom X, decreased slightly with the increase of atomic number (from Sc to Zn 3d elements and from Y to Cd 4d elements). It is very interesting that Bader charge difference of metal atom changed with atomic number. When Sc, Ti, V, Y or Zr was added into the Al(111)/SiC(0001) interface, it lost charges just like the Al atom, but when Mn, Fe, Co, Ni, Cu, Zn, Mo, Tc, Ru, Rh, Pd, Ag or Cd atom was added to the interface, it gained electrons, some of which came from nonmetal atoms and some from Al atoms. When Mn, Fe, Co, Ni, Cu, Zn, Mo, Tc, Ru, Rh, Pd, Ag or Cd atom is added to the interface, there was charge transfer between doped atoms and Al atoms. These results indicated that the transition metal atoms not only formed covalent bonds with nonmetallic C (Si) atoms at the interface, but also formed metal bonds with Al atoms.

Especially, when a Co atom was doped into the C-terminated Al(111)/SiC(0001) interface, it obtained electrons not only from the C atom, but also from the Al atom. In this way, the introduction of Co atoms promoted the formation of not only strong Co–C bonds, but also stronger Al–C bonds at the interface. Therefore, the adhesion energy of the C-terminated Al-Co (111)/SiC (0001) interface was higher than that of the pristine C-terminated Al (111)/SiC (0001) interface. Although there was more charge transfer at the C-terminated interface of Tc-Al(111)/SiC(0001), Ru-Al(111)/SiC(0001) and Rh-Al(111)/SiC(0001), the radius of Tc, Ru, Rh was too large to form stronger covalent bonds. Thus, the introduction of Tc, Ru or Rh into the C-terminated Al(111)/SiC(0001) interface could not improve the interfacial adhesion energy.

The strength of Si–Al bond –smaller than that of C–Al bond. When Mn, Fe, Co, Ni, Cu, Zn, Tc, Ru, Rh, Pd or Ag was added into the Si-terminated Al(111)/SiC(0001) interface, more charge transfer occurred between the doping atom and other atoms. A stronger covalent bond was formed between the Si atom and the doping atom. Therefore, the introduction of Mn, Fe, Co, Ni, Cu, Zn, Tc, Ru, Rh, Pd or Ag into the Si-terminated Al(111)/SiC(0001) interface could improve the interfacial adhesion energy. Because of lower surface energy of the Si-terminated SiC(0001), the Si-terminated Al(111)/SiC(0001) interface was more prone to existing. Therefore, adding the transition metal elements into SiC particle reinforced aluminum matrix composites was mainly used to improve the adhesion energy of Si-terminated interface, and then improve the mechanical properties of the composites.

## 4. Conclusions

The effects of 20 transition elements doping on the interfacial adhesion and electronic structure of Al(111)/6H-SiC(0001) interfaces have been studied by First-principles methods in this paper. The main conclusions are summarized as follows: (1) For the pristine Al(111)/6H-SiC(0001) interface, covalent bonds are formed at both C-terminated and Si-terminated interfaces. According to Bader’s charge analysis, there is more charge transfer between C and Al at the C-terminated interface, which leads to higher adhesion energy. (2) For the C-terminated Al(111)/6H-SiC(0001) interface, the adhesion energy of the interface can be improved only when Co is doped at the interface. The strength of covalent bond between transition metal atom and C atom is weaker than that of C–Al bond. This may be attributed to the larger atomic radius of transition metal atoms. (3) For the Si-terminated Al(111)/6H-SiC(0001) interface, when Mn, Fe, Co, Ni, Cu, Zn, Tc, Ru, Rh, Pd or Ag is doped at the interface, the adhesion energy of the interface can be improved. It is mainly due to the formation of stronger Si–X bonds at the interface. The doped transition metal atom not only forms a strong covalent bond with the Si atom, but also promote more charge transfer between Al atoms and Si atoms, forming stronger Si–Al bonds. These results are helpful to understand the mechanism of Al/SiC interfacial wettability and adhesion.

## Figures and Tables

**Table 1:** Bond length (Å) and adhesion energy $E_{ad}$ (J/m^2^) at the C-terminated Al-X (111)/6H-SiC (0001) interface.

Interfaces	Doping Elements	C-Al (Å)	C-X (Å)	$E_{ad}\;(J/m^{2})$	Doping Elements	C-Al (Å)	C-X (Å)	$E_{ad}(J/m^{2})$
C-terminated	Sc	2.03	2.12	3.14	Y	2.06	2.25	3.18
	Ti	2.01	2.07	3.64	Zr	2.03	2.22	3.39
	V	1.99	2.05	3.72	Nb	2	2.2	3.47
	Cr	1.98	2.04	3.78	Mo	1.98	2.18	3.63
	Mn	1.97	2.02	3.84	Tc	1.97	2.17	3.83
	Fe	1.96	2.01	3.9	Ru	1.96	2.16	3.91
	Co	1.95	2	3.99	Rh	1.96	2.17	3.85
	Ni	1.96	2.02	3.92	Pd	1.97	2.18	3.73
	Cu	1.97	2.03	3.84	Ag	1.98	2.2	3.62
	Zn	1.98	2.04	3.79	Cd	1.99	2.21	3.5
	Free	1.99	-	3.9	-	-	-	-

**Table 2:** Bond length (Å) and adhesion energy $E_{ad}$ J/m^2^) at the Si-terminated Al-X (111)/6H-SiC (0001) interface.

Interfaces	Doping Elements	Si-Al (Å)	Si-X (Å)	$E_{ad}\;(J/m^{2})$	Doping Elements	Si-Al (Å)	Si-X (Å)	$E_{ad}\;(J/m^{2})$
Si-terminated	Sc	2.6	2.68	2.51	Y	2.62	2.78	2.49
	Ti	2.56	2.63	2.66	Zr	2.59	2.72	2.55
	V	2.53	2.6	2.8	Nb	2.55	2.68	2.66
	Cr	2.52	2.56	2.88	Mo	2.52	2.65	2.85
	Mn	2.51	2.53	3.05	Tc	2.5	2.61	3.03
	Fe	2.49	2.51	3.21	Ru	2.49	2.58	3.13
	Co	2.46	2.48	3.2	Rh	2.48	2.55	3.14
	Ni	2.47	2.5	3.17	Pd	2.49	2.54	3.08
	Cu	2.5	2.53	3.07	Ag	2.52	2.55	2.97
	Zn	2.52	2.58	3.03	Cd	2.54	2.57	2.8
	Free	2.53	-	2.93	-	-	-	-

## Data Availability

Data sharing is not applicable to this article.
